# Tetramethylpyrazine modulates airway inflammation and remodeling in bronchial asthma: A review

**DOI:** 10.1016/j.clinsp.2025.100768

**Published:** 2025-09-22

**Authors:** Zichao Han, Fei Yu, Zhen Ding, Qinghua Liu, Xiangyi Zheng, Lijuan Zhang, Dong Li, Chao Wang

**Affiliations:** a Zibo Traditional Chinese Medicine Hospital, Zibo 255300, Shandong, China; b Zibo Central Hospital, Zibo 255022, Shandong, China

**Keywords:** Tetramethylpyrazine, Bronchial asthma, Immune homeostasis, Airway remodeling

## Abstract

Bronchial Asthma, characterized by chronic airway inflammation and remodeling, poses significant clinical challenges. Long-term use of conventional therapies often leads to metabolic complications and drug resistance. The bioactive alkaloid Tetramethylpyrazine, derived from *Ligusticum chuanxiong* Hort. (Apiaceae), demonstrates multi-target therapeutic potential through pathway modulation. TMP restores Th1/Th2 and Th17/Treg immune balance via Gαs/cAMP/PKA and AMPK/NF-κB signaling while suppressing ORMDL3-mediated collagen deposition. Clinical evidence demonstrates effective symptom alleviation with minimal adverse effects, establishing TMP as a promising complementary strategy for asthma management.

## Highlights

• Tetramethylpyrazine (TMP) can restore the balance of Th1/Th2 and Th17/Treg in asthma.
• TMP can regulate signaling pathways such as Gαs/cAMP/PKA and AMPK/NF-κB.
• TMP inhibits airway remodeling through TGF-β and ORMDL3.
• TMP can reduce vascular remodeling by inhibiting factors like VEGF.
• This review emphasizes that TMP is a promising complementary therapy for asthma.

## Introduction

BA is a chronic airway disorder driven by Th2-mediated inflammation, clinically manifesting as bronchial hyperresponsiveness and variable airflow obstruction.^1^ Its prevalence is notably elevated in pediatric populations,^2^ with global incidence rising due to environmental pollution, lifestyle shifts, and aging demographics. Current projections estimate approximately 400 million cases worldwide by 2025,^3^^,^^4^ posing substantial health burdens and socioeconomic challenges. The Global Initiative for Asthma recommends ICS-LABA combination therapy as first-line treatment. Although effective against acute inflammation and bronchoconstriction,^5^ long-term use causes adverse effects ranging from oral candidiasis to osteoporosis.^6^ Safety concerns, particularly β_2_-adrenoceptor-mediated cardiotoxicity, have intensified demand for novel therapeutics. Recent studies reveal TMP's unique ability to concurrently target the inflammation-immunity-remodeling axis.^7^ This review synthesizes TMP's anti-asthma mechanisms, deciphers its multi-target networks, and proposes integrative strategies to overcome current therapeutic limitations.

### TMP: Source and pharmacodynamics

**Fig. 1:**
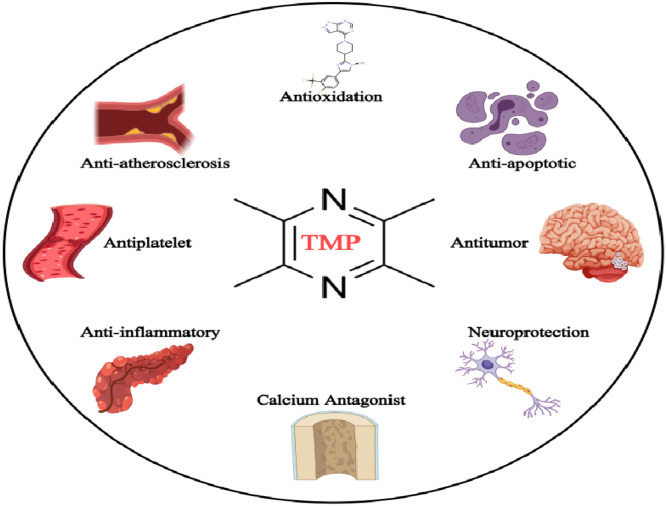
Pharmacological Effects of TMP (Tetramethylpyrazine).

*Ligusticum chuanxiong* Hort. (Apiaceae), a canonical herb in Traditional Chinese Medicine, was first documented in *Shennong Bencao Jing* (circa 200 BCE) for promoting blood circulation and relieving pain. Modern studies confirm its circulatory benefits derive from enhancing microcirculation and inhibiting platelet aggregation.^8^ The rhizome harbors diverse bioactive compounds: phthalides, phenolic acids, polysaccharides, and alkaloids.^9^ Among these, TMP stands out as the principal alkaloid,^10^ exhibiting multi-target effects including anti-inflammatory, antitumor, neuroprotective, and antioxidant activities^11^, ^12^, ^13^, ^14^, ^15^ (Fig. 1). Although clinically used for cardiovascular and neurodegenerative diseases,^16^, ^17^, ^18^ TMP shows exceptional potential in respiratory disorders by simultaneously suppressing airway inflammation and remodeling in asthma.^19^^,^^20^

### Regulation of inflammatory cytokines and immune balance

#### Restoring Th1/Th2 balance

**Fig. 2:**
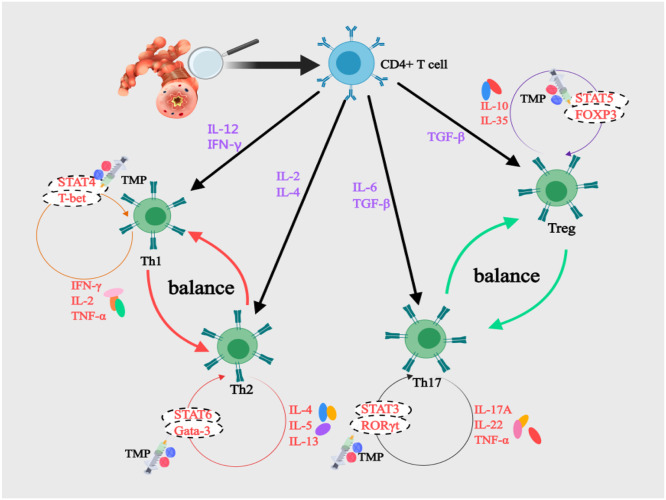
Mechanisms of Th1/Th2 and Th17/Treg Balance in BA and the Intervention of TMP. FOXP3, Forkhead Box Protein P3; Gata, GATA binding protein; IFN-γ, Interferon-γ; IL, Interleukin; RORγt, Retinoic acid Receptor-related Orphan Receptor γt; STAT, Signal Transducer and Activator of Transcription; T-bet, T-box expressed in T-cells; TGF-β, Transforming Growth Factor-Beta; Th, T-Helper; TMP, Tetramethylpyrazine; TNF-α, Tumor Necrosis Factor-α; Treg, Regulatory-T.

Functioning as pivotal regulators of adaptive immunity, CD4^+^T-lymphocytes undergo differentiation into distinct Th (Th1, Th2, Th17) cells and Treg cells.^21^ In asthma, Th1/Th2 and Th17/Treg imbalance drives immunopathology (Fig. 2). IL-12 and IFN-γ activate STAT4/T-bet signaling to induce Th1 differentiation,^22^ enhancing macrophage phagocytosis for antimicrobial defense.^23^ IFN-γ further amplifies Th1 responses via STAT1-mediated T-bet upregulation, which suppresses IL-4 activity and establishes early protective immunity.^24^^,^^25^ Conversely, IL-4 activates STAT6/GATA-3 to polarize Th2 cells, triggering excessive IL-5/IL-13 secretion that fuels allergic responses.^26^ This cascade elevates IgE levels and recruits eosinophils through CCL11/CCL24, perpetuating type 2 inflammation.^27^ Asthma pathogenesis thus hinges on Th1/Th2 imbalance sustained by type 2 cytokines.^28^ TMP complements glucocorticoids by restoring immune equilibrium.^7^ In OVA-sensitized murine models, Xiong et al.^29^ demonstrated TMP's dual immunomodulatory effects. Administration of 80 mg/kg TMP significantly suppressed eosinophil infiltration and downregulated Th2-associated markers IL-4 and GATA-3 (*p* < 0.05). Concurrently, this treatment upregulated Th1-related factors IFN-γ and T-bet (*p* < 0.05) through coordinated T-bet/GATA-3 ratio modulation. Yan et al.^30^ conducted a clinical trial evaluating TMP as adjunctive therapy in pediatric asthma. Patients receiving 2.5‒5 mg/kg/d TMP for 10 days exhibited significant immunological shifts: serum IL-4 levels decreased from 56.30 ± 14.32 pg/mL to 38.24 ± 12.10 pg/mL (*p* < 0.05). Simultaneously, IFN-γ concentrations increased from 16.58 ± 6.25 ng/mL to 28.42 ± 10.37 ng/mL (*p* < 0.05). This bidirectional cytokine modulation effectively reversed Th1-Th2 clonal drift.

The transmembrane glycoprotein CD44 regulates cellular adhesion, migration, and signaling through interactions with ECM components such as hyaluronic acid.^31^ In asthma, CD44 overexpression facilitates eosinophil and neutrophil adhesion, driving airway infiltration and Th2-polarized inflammation.^32^ Kumar et al.^33^ found that CD44-knockout mice exposed to house dust mites and cigarette smoke showed significantly reduced inflammatory cell counts (*p* < 0.05) and suppressed T2 cytokine production. Compared to wild-type controls, these mice also exhibited fewer peribronchial eosinophils and mucus cells, confirming CD44 inhibition as an effective anti-inflammatory strategy. Li et al.^34^ revealed TMP's dose-dependent suppression of CD44 expression in asthma models. High-dose TMP (80 mg/kg) demonstrated superior efficacy to both lower-dose TMP (40 mg/kg) and dexamethasone (0.5 mg/kg) in mitigating pathological features.

#### Restoring Th17/Treg balance

The Th17/Treg balance is a pivotal axis in asthma immunopathology, paralleling the importance of Th1/Th2 equilibrium. Th17 differentiation is driven by STAT3/RORγt signaling, contrasting with Treg development via STAT5-dependent FOXP3 activation.^35^ Th17 cells exacerbate inflammation by secreting IL-17A/IL-22/TNF-α, which recruit neutrophils and induce bronchoconstriction.^36^ Conversely, Tregs sustain immune tolerance through IL-10/IL-35 secretion, suppressing effector T-cell responses.^37^ Li et al.^38^ identified elevated serum IL-17 levels in acute asthma patients versus stable patients and healthy controls (*p* < 0.05). OVA-sensitized models mirrored this trend, showing increased IL-17 production (*p* < 0.05) with Th17/Treg imbalance driving disease progression. Moreover, Ji et al.^39^ revealed a critical limitation of dexamethasone: while effectively suppressing Th2 cytokines (IL-4/IL-5), it fails to inhibit Th17-derived IL-17. In contrast, TMP demonstrated dual immunomodulatory capacity by simultaneously blocking both Th2 and Th17 cytokine production while enhancing IL-10 secretion, albeit without altering FOXP3 expression patterns. This unique immunomodulatory profile reduces eosinophil/neutrophil infiltration and corrects Th17/Treg imbalance in allergic airway inflammation. Supporting evidence from Pan's team^40^ observed that TMP administration (1 mL/kg) upregulated both FOXP3 gene and protein expression in cerebral ischemia models. This cross-model evidence further supports TMP's regulatory capacity over the FOXP3/RORγt axis.

#### Modulating the Gαs/cAMP/PKA pathway

**Fig. 3:**
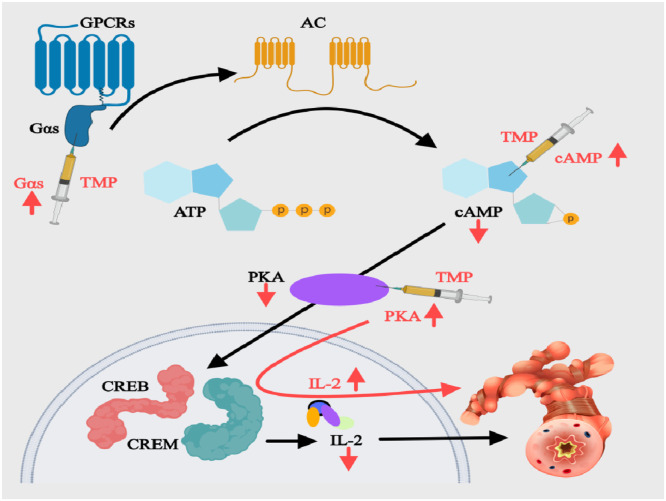
Mechanisms of the Gαs/cAMP/PKA Pathway in BA and the Intervention of TMP. AC, Adenylate Cyclase; ATP, Adenosine Triphosphate; Camp, cyclic Adenosine Monophosphate; CREB, cAMP-Response Element Binding; CREM, cAMP-Response Element Modulating; GPCRs, G Protein-Coupled Receptors; IL, Interleukin; PKA, Protein Kinase A; TMP, Tetramethylpyrazine.

The Gαs/cAMP/PKA axis is a critical signaling pathway disrupted in asthma. As a GPCRs component, Gαs dysfunction promotes airway inflammation (Fig. 3). Upon activation, Gαs dissociates from Gβγ to stimulate AC, converting ATP to Camp.^41^ This second messenger (cAMP) activates PKA, inducing phosphorylation of CREB/CREM transcription factors to maintain IL-2 production, T-cell proliferation, and anti-inflammatory mediator expression.^42^ In asthma, this pathway is suppressed, marked by reduced cAMP, impaired PKA activity, and inflammatory cell influx.^43^ Wang et al.^44^ demonstrated that daily 40 mg/kg TMP administration in OVA-induced asthmatic rats not only upregulated pulmonary Gαs expression (*p* < 0.01) but also restored cAMP levels and enhanced PKA activity (*p* < 0.01), collectively reactivating the impaired Gαs/cAMP/PKA pathway. This pathway reactivation significantly suppressed key Th2 cytokines ‒ TNF-α, IL-4, and IL-5 ‒ while improving immune balance (*p* < 0.01). PDE is a class of enzymes that hydrolyze cAMP and terminate its biochemical actions. Experimental validation shows that 40 mg/kg TMP administration suppresses PDE expression at both mRNA and protein levels. This dual inhibition of cAMP hydrolysis effectively attenuates airway hyperresponsiveness, as evidenced in preclinical models.^45^ TMP demonstrates partial β_2_-AR agonism, activating the cAMP/PKA pathway to induce bronchodilation comparable to salbutamol. Crucially, this effect occurs without the tachycardia or other adverse effects linked to conventional β-agonists.^46^

#### Modulating the AMPK/NF-κB pathway

**Fig. 4:**
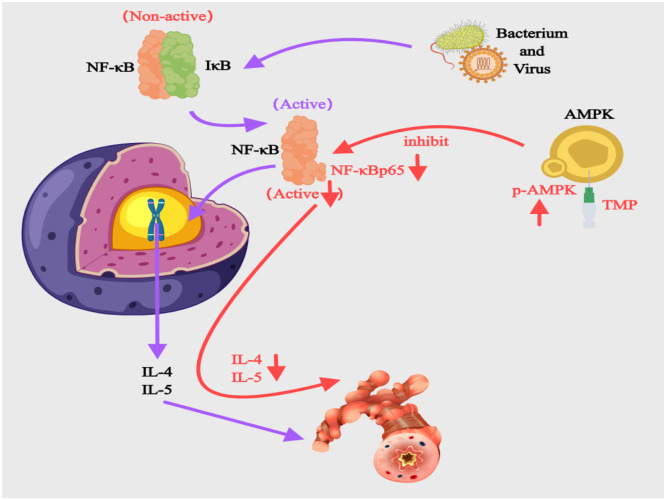
Mechanisms of the AMPK/NF-κB Pathway in BA and the Intervention of TMP. AMPK, AMP-activated Protein Kinase; IL, Interleukin; NF-κB, Nuclear Factor-κB; TMP, Tetramethylpyrazine.

The transcription factor NF-κB drives chronic inflammation in asthma when abnormally activated (Fig. 4). At rest, NF-κB remains inactive by binding to IκB. External triggers like bacteria or viruses activate TLRs, leading to IκB kinase-mediated IκBα phosphorylation and degradation. This exposes nuclear localization signals, allowing NF-κB to enter the nucleus and bind κB sites on the IL4 promoter, triggering excessive Th2 cytokine production.^47^ AMPK acts as a key regulator connecting energy metabolism and inflammation. It blocks NF-κB activity through two main methods. First, it phosphorylates IκB kinase to disable its function. Second, it increases SIRT1 production to remove acetyl groups from p65. Together, these actions lower inflammatory molecules and protect against asthma.^48^ In OVA-induced asthmatic mice, Xu et al.^49^ showed that intraperitoneal TMP (100‒200 mg/kg) boosted AMPK phosphorylation and increased p-AMPK/AMPK ratios in bronchial cells while suppressing NF-κB p65 protein. This combined action effectively controlled airway inflammation. Liu et al.^19^ additionally, confirmed TMP's suppression of NF-κB signaling, which lowers IL-1β and TNF-α levels to ease airway inflammation in neutrophilic asthma models. These combined results highlight TMP's promise for developing new asthma treatments.

#### Modulating the MAPK/ERK pathway

**Fig. 5:**
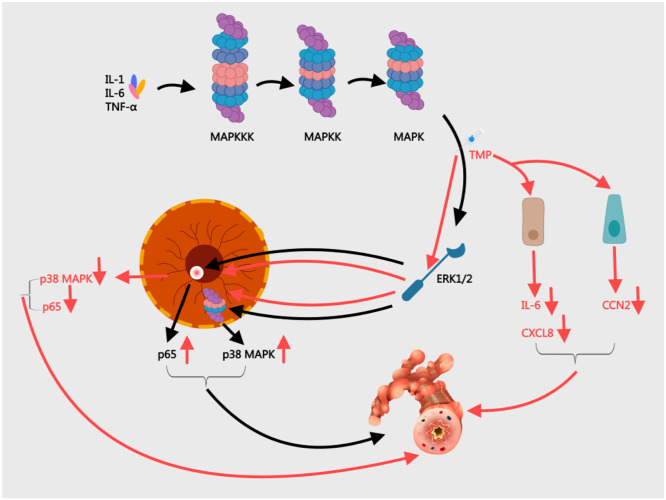
Mechanisms of the MAPK/ERK Pathway in BA and the Intervention of TMP. ERK, Extracellular Regulated Protein Kinases; CCN2 (CTGF), Connective Tissue Growth Factor; CXCL8, IL (Interleukin)-8; MAPK, Mitogen-Activated Protein Kinase; TMP, Tetramethylpyrazine; TNF, Tumor Necrosis Factor-α.

MAPK enzymes respond to various triggers (e.g., IL-1, IL-6) by activating serine/threonine kinases. In asthma, abnormal p38α MAPK activation drives disease progression, transmitting extracellular signals to the nucleus to alter gene expression and cellular responses.^50^ Liang et al.^51^ found OVA sensitization increased phosphorylated p38 MAPK and nuclear p65 levels in murine lungs while reducing oxidative stress markers MDA and GSH-Px (*p* < 0.05). The p38 MAPK pathway activates ERK1/2 through a three-tier signaling cascade (MAPKKK→MAPKK →MAPK), critically amplifying airway inflammation^52^ (Fig. 5). Mei et al.^53^ discovery that daily 80 mg/kg TMP treatment reduced p38 MAPK staining intensity in asthmatic mice from 0.217 ± 0.014 to 0.156 ± 0.003 (*p* < 0.05), effectively alleviating bronchial smooth muscle spasms, airway narrowing, and inflammatory cell infiltration. Wei's team^54^ confirmed the TMP suppresses p38 MAPK signaling to decrease neutrophils, lymphocytes, and eosinophils while lowering IL-4/IL-5 and related chemokines in asthma models. Beyond inflammation control, p38 MAPK contributes to airway remodeling. Inhibiting this pathway reduces fibroblast-derived IL-6/IL-8 and bronchial cell CTGF production, countering fibrosis and structural changes.^55^^,^^56^

In asthmatic inflammation, pro-inflammatory factors like IL-1β and TNF-α activate the MAPK/ERK pathway, rapidly inducing transcription of the proto-oncogene c-fos. The c-fos protein then combines with JNK to form the AP-1 complex, which drives expression of downstream inflammatory mediators (COX-2, MMPs) that amplify airway inflammation and hyperresponsiveness.^57^ Animal studies^58^ demonstrated that TMP treatment significantly reduced lung c-fos levels in asthmatic rats, with mRNA decreasing from 0.40 ± 0.06 to 0.19 ± 0.05 and protein from 0.56±0.07 to 0.31 ± 0.12 (*p* < 0.01). By suppressing AP-1 complex formation, TMP further lowered IL-8 and TNF-α release, confirming its therapeutic effect on airway inflammation. In simulated weightlessness models using tail suspension,^59^ TMP modulated ERK1/2 signaling to suppress excessive c-fos expression (0.14 ± 0.01 in TMP-treated vs. 0.16 ± 0.01 in tail-suspended controls; *p* < 0.05). This intervention reduced abnormal proliferation of vascular endothelial and smooth muscle cells; effectively mitigating lung tissue damage caused by simulated microgravity.

#### Additional mechanisms

SCF exacerbates asthma by activating mast cells and modulating immune responses. It promotes mast cell survival, proliferation, and activation, driving IL-13 secretion that amplifies airway inflammation and hyperreactivity.^60^ Furthermore, SCF enhances mast cell migration to inflammatory sites via c-Kit receptor activation.^61^ Normal mice-maintained serum SCF levels at 48.6 ± 11.2 pmoL/L, which surged to 114.9 ± 27.3 pmoL/L (*p* < 0.01) after 1 % OVA challenge. TMP dose-dependently reversed this increase, reducing levels to 70.6 ± 7.9 pmoL/L with 40 mg/kg and near-baseline 51.4 ± 8.1 pmoL/L at 80 mg/kg.^62^ The observed SCF reduction implies TMP's regulatory effect on this pathway, warranting further investigation into its therapeutic potential for asthma treatment.

P-selectin and E-selectin, as adhesion molecules, drive airway inflammation by mediating leukocyte-endothelial adhesion and migration. In patients experiencing acute asthma exacerbation, P-selectin levels are slightly elevated but return to baseline within 36 months.^63^ This sustained elevation critically exacerbates eosinophil infiltration.^64^ E-selectin, mainly endothelial-derived, promotes leukocyte rolling and firm adhesion, facilitating transendothelial migration. This process releases histamine and leukotrienes, causing bronchoconstriction, mucus hypersecretion, and chronic airway remodeling.^65^ Preclinical studies in OVA-sensitized rats revealed that 80 mg/kg TMP significantly reduced serum P-selectin levels from 72.39±3.78 ng/L to 24.17±1.98 ng/L (*p* < 0.05). This reduction outperformed both 40 mg/kg TMP alone and the combination of 40 mg/kg TMP with 0.25 mg/kg dexamethasone. Further analysis showed prolonged 80 mg/kg TMP administration achieved efficacy comparable to 0.5 mg/kg dexamethasone.^66^ Clinically, intravenous TMP (3‒5 mg/kg) significantly lowered serum P-selectin in pediatric asthma patients (*p* < 0.01), with 95 % therapeutic efficacy surpassing the 70 % observed with glucocorticoids (*p* < 0.05).^67^ Higher doses (40‒80 mg/day IV) also modulated soluble E-selectin and improved lung function.^68^ These findings collectively reveal TMP’s dual mechanism: disrupting P/E-selectin-mediated leukocyte adhesion/migration and rebalancing inflammatory networks to suppress airway pathology.

Asthma often involves platelet activation and aggregation. TMP counters this by lowering intracellular calcium levels in platelets, thereby reducing their activation, aggregation, and adhesion. This suppression decreases lymphocyte and eosinophil mobilization while significantly curtailing IL-5 and IL-13 production in asthmatic lungs, demonstrating potent immunomodulatory and anti-inflammatory effects.^69^

### Suppressing airway remodeling

Airway remodeling represents a core pathological feature of chronic asthma, characterized by irreversible structural alterations. Key changes encompass epithelial damage, thickened basement membranes, smooth muscle overgrowth, goblet cell metaplasia, excessive blood vessel formation, and abnormal ECM buildup.^70^ Chronic inflammation drives EMT, causing epithelial cells to lose polarity and transform into fibroblasts. This process promotes excessive type I/III collagen deposition beneath the basement membrane, leading to fibrosis and airway narrowing. Concurrently, TGF-β and VEGF activate fibroblasts/myofibroblasts to overproduce fibronectin while inhibiting MMP activity, disrupting ECM homeostasis.^71^ Aberrant activation of PDGF and EGF pathways drives ASMC proliferation and hypertrophy, enhancing contractility and pro-fibrotic mediator secretion. This exacerbates airway hyperresponsiveness through structural and functional alterations.^72^ Concurrently, VEGF-mediated angiogenesis and NGF overexpression worsen airway edema and neuronal hypersensitivity, creating a self-perpetuating inflammation-remodeling cycle.^73^ These pathological cascades collectively result in irreversible airway narrowing, glucocorticoid resistance, and progressive lung function deterioration.

#### Modulating TGF-β/Smad signaling, and MMP-9/TIMP-1 balance

**Fig. 6:**
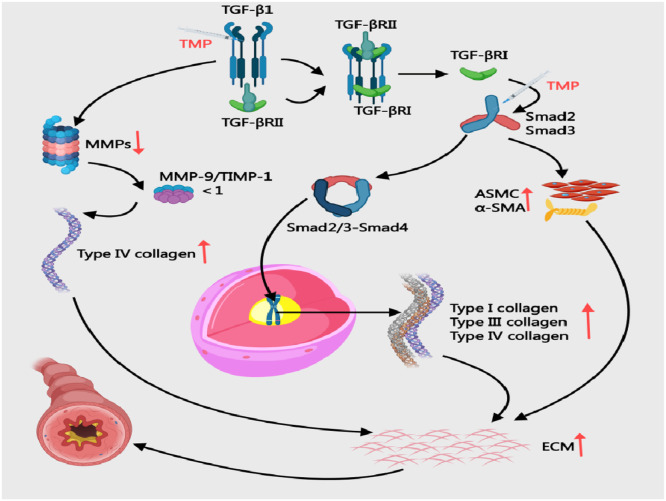
Mechanisms of Collagen Deposition in BA and the Intervention of TMP. ASMC, Airway Smooth Muscle Cells; ECM, Extracellular Matrix; TGF-β1, Transforming Growth Factor-beta; TIMP, Tissue Inhibitor of Metalloproteinases; MMP-9, Matrix Metallopeptidase 9; TMP, Tetramethylpyrazine; α-SMA, α-Smooth Muscle Actin.

Collagens III/IV critically drive asthma airway remodeling by altering ECM structure/function, contributing to wall thickening and fibrosis.^74^ The TGF-β/Smad signaling pathway drives asthmatic airway remodeling through multifaceted mechanisms. Upon activation, TGF-β1 binds to TGF-βRⅡ and phosphorylates TGF-βRⅠ, initiating Smad 2/3 phosphorylation. These phosphorylated Smad proteins combine with Smad4 to form transcriptional complexes that migrate into the nucleus, directly upregulating collagen types I/III/IV and fibronectin expression. This molecular cascade promotes excessive ECM deposition and basement membrane thickening.^75^^,^^76^ Simultaneously, TGF-β1 suppresses MMPs production, impairing ECM degradation capacity.^77^ Additionally, the pathway induces EMT and activates fibroblasts, which stimulate ASMC proliferation and α-SMA overexpression. These cellular changes collectively exacerbate airway constriction and airway hyperresponsiveness^78^ (Fig. 6). In OVA/Al (OH)_3_-sensitized mice, 80 mg/kg TMP reduced TGF-β1 (0.456 ± 0.073 vs. 0.656 ± 0.049) and Smad2 (0.370 ± 0.042 vs. 0.591 ± 0.074) while increasing Smad7 (0.279 ± 0.056 vs. 0.137 ± 0.055; *p* < 0.05), effectively curbing collagen deposition.^79^ PKB and ERK proteins function as downstream effectors of the TGF-β/Smad pathway, indirectly modulated by this signaling cascade to drive airway remodeling.^80^ In CVA rats, 1.05 mg/kg TMP surpassed 32.5 mg/kg montelukast in suppressing TGF-β/PKB/ERK (*p* < 0.05), indicating pathway modulation.^81^ In OVA-sensitised asthmatic rats, intraperitoneal injection of 5 mg TMP resulted in lower expression of both MMP-9 and TIMP-1 compared to the asthma model group. This was accompanied by reduced airway smooth muscle thickness, decreased type IV collagen deposition, and attenuated progression of airway remodelling.^82^

#### Inhibition of EMT progression

**Fig. 7:**
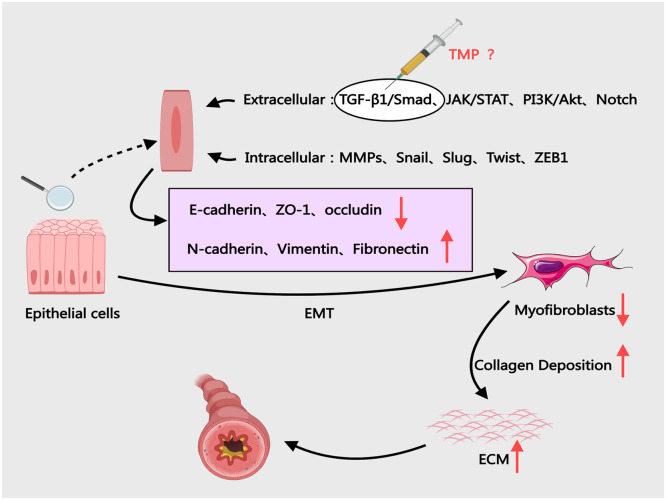
The Role of EMT in BA and the Potential Effects of TMP. ECM, Extracellular Matrix; EMT, Epithelial-mesenchymal Transition; JAK, Janus Kinase; STAT, Signal Transducer and Activator of Transcription; TGF-β, Transforming Growth Factor-beta; MMPs, Matrix Metallopeptidases; TMP, Tetramethylpyrazine.

The airway epithelium forms a continuous protective barrier, with intercellular junctions maintaining structural integrity.^83^ Post-injury, adhesion junction remodeling mediated by VEGF, TGF-β1, and FGF facilitates epithelial rearrangement and repair.^84^ Normally, myofibroblasts derived from EMT undergo apoptosis post-repair, leaving residual collagen for physiological ECM remodeling.^85^ In asthma, pathological EMT transforms polarized epithelial cells into migratory mesenchymal cells, driving airway remodeling and chronic inflammation^86^ (Fig. 7). Beyond the TGF-β1/Smad signaling pathway, additional pathways, including JAK/STAT, PI3K/PKB, and Notch signaling, are implicated in driving epithelial-mesenchymal transition during airway remodeling.^87^ IL-6 and IFN-γ activate the JAK/STAT pathway through receptor binding. This activation triggers STAT phosphorylation, leading to nuclear translocation of dimerized transcription factors. These factors reprogram gene networks that regulate cellular proliferation, apoptosis, and inflammatory responses ‒ all critical drivers of airway remodeling.^88^ Experimental evidence demonstrates that Ligusticum chuanxiong-containing Taohong Siwu Decoction attenuates pulmonary fibrosis by reducing TGF-β1, TNF-α, and α-SMA expression (*p* < 0.01), effectively mitigating alveolar EMT.^89^ However, current evidence gaps persist regarding TMP's capacity to modulate PI3K/PKB or Notch signaling in human asthmatic EMT processes.

#### Inhibition of ORMDL3 gene expression

**Fig. 8:**
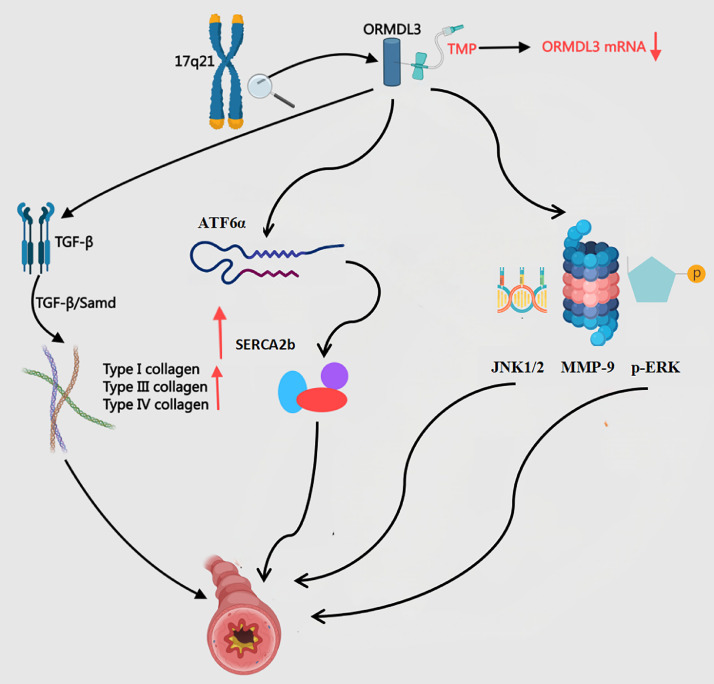
The Mechanism of ORMDL3 in BA and the Intervention of TMP. ATF6α, Activating Transcription Factor 6 alpha; p-ERK, p-Extracellular Regulated Protein Kinases; JNK, c-Jun N-terminal Kinase; SERCA2b, Sarco/Endoplasmic Reticulum Ca2+-ATPase 2b; TGF-β, Transforming Growth Factor-Beta; MMP-9, Matrix Metallopeptidase-9; ORMDL3, Orosomucoid-Like-3; TMP, Tetramethylpyrazine.

The endoplasmic reticulum transmembrane protein ORMDL3, encoded by the chromosomal locus 17q21, functions as a genetic susceptibility factor for bronchial asthma and drives airway remodeling through multi-mechanistic pathways^90^ (Fig. 8). This protein activates the UPR, inducing TGF-β release from airway epithelial cells to promote fibroblast activation and collagen deposition, thereby accelerating airway fibrosis.^91^ ORMDL3 activates the ATF6α pathway within the unfolded protein response (UPR), upregulates SERCA2b expression, and subsequently induces the production of multiple pro-inflammatory and pro-remodelling factors in human bronchial epithelial cells, thereby directly linking endoplasmic reticulum stress to the process of airway remodelling in asthma.^92^ ORMDL3 promotes thickening and remodelling of the airway wall in asthma patients by activating the p-ERK/MMP-9 pathway and upregulating the expression of p-ERK and MMP-9.^93^ Furthermore, ORMDL3, which is highly expressed in asthma, exacerbates airway inflammation and remodelling by activating the JNK1/2–MMP-9 pathway, representing a potential novel therapeutic target for asthma treatment.^94^ Li et al.^95^ found that ORMDL3 overexpression in asthmatic mouse lungs boosts 16HBE-14° cell migration. This genetic alteration also activates NF-κB and MAPK/ERK phosphorylation while undermining dexamethasone's therapeutic efficacy. Such molecular resistance clarifies why glucocorticoids ‒ despite being first-line asthma therapies ‒ fail to prevent disease relapse. TMP administered at 400 μg/L significantly reduces ORMDL3 mRNA and protein levels in human bronchial epithelial cells (*p* < 0.01). This suppression effectively slows airway remodeling progression.^96^ Compared to glucocorticoids, TMP demonstrates longer-lasting therapeutic benefits.

#### Inhibiting vascular remodeling

##### Suppressing VEGF levels

**Fig. 9:**
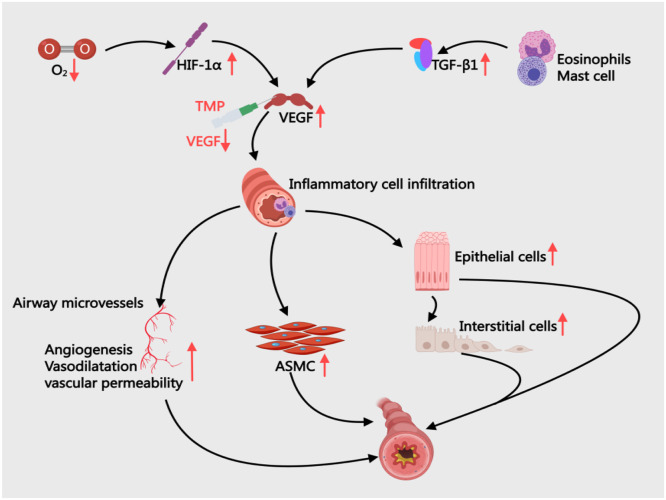
The Mechanism of VEGF in BA and the Intervention of TMP. ASMC, Airway Smooth Muscle Cells; HIF-1α, Hypoxia-Inducible Factor-1; TGF-β, Transforming Growth Factor-beta; TMP, Tetramethylpyrazine; VEGF, Vascular Endothelial Growth Factor.

VEGF plays a central role in airway remodeling (Fig. 9). Its overexpression stimulates vascular endothelial cell proliferation, increases microvascular density, and exacerbates airway wall edema, inflammatory infiltration, and hyperresponsiveness.^97^ Elevated VEGF correlates with key remodeling markers such as smooth muscle thickening and collagen deposition. Through activating downstream pathways like KDR, VEGF promotes ASMC proliferation and accumulation of matrix components (collagens I/III), leading to airway wall thickening and luminal narrowing.^98^ Additionally, VEGF synergizes with Th2 cytokines (IL-4/IL-5) to amplify eosinophilic infiltration, worsening airway inflammation and fibrosis.^99^ Nasser et al.^100^ reported significantly higher serum VEGF-A levels in asthmatic children compared to healthy controls (*p* < 0.05) via ELISA. In OVA-sensitized rats, Yan et al.^101^ observed that 2 mg TMP injections reduced airway VEGF (TMP: 13.27 ± 5.72 vs. BA: 18.59 ± 6.32 pg/min) and iNOS (TMP: 15.79 ± 6.61 vs. BA: 22.23 ± 7.91 pg/min), effectively suppressing remodeling. Both VEGF and iNOS levels positively correlated with WA/Pi and SMC-A/Pi ratios, confirming their roles in structural changes.

##### Inhibiting HUVECs function

HUVECs derived from neonatal umbilical veins play dual roles in vascular remodeling associated with asthma. During asthmatic pathophysiology, IL-8-mediated pro-angiogenic activity enhances HUVECs proliferation, migration, and permeability. The abnormal vascular growth and structural remodeling driven by these changes strongly correlate with persistent Th2/Th17 inflammatory responses.^102^ Microenvironmental homeostasis critically regulates HUVECs phenotypes. Studies by Faiz et al.^103^ demonstrate that removing inflammatory stimuli restores physiological ECM function in asthmatic conditions. This normalized ECM maintains HUVECs quiescence and vasodilatory capacity without inducing pathological angiogenesis. MSCs modulate HUVECs through dual mechanisms. First, Paracrine release of VEGF and HGF stimulates HUVEC proliferation and vascular regeneration. Second, Secretion of IL-10 and TGF-β suppresses Th2/Th17 polarization, blocking IL-5/IL-17A-induced endothelial activation to preserve vascular homeostasis.^104^ Thus, HUVECs operate within a microenvironment-dependent “dual regulatory network” ‒ exacerbating vascular remodeling under inflammation while promoting repair via MSC interactions during homeostasis. Although TMP lacks direct inhibitory effects on HUVECs, its broad anti-inflammatory and immunomodulatory properties may indirectly attenuate pathological HUVECs activation by mimicking MSC-mediated protective mechanisms. However, this hypothesis remains under investigation, requiring further experimental validation and clinical evidence to confirm its scientific validity and generalizability.

## Summary

As an alkaloid possessing potent anti-inflammatory and immunomodulatory properties, TMP demonstrates multifaceted therapeutic potential for asthma management. Research reveals TMP's ability to intervene in asthmatic pathology through multi-target mechanisms. It precisely restores immune balance by normalizing Th1/Th2 and Th17/Treg equilibrium, thereby correcting inflammation caused by immune dysregulation. TMP concurrently inhibits activation of critical signaling pathways ‒ including Gαs/cAMP/PKA, AMPK/NF-κB, and MAPK/ERK cascades ‒ to block inflammatory signal transduction and suppress inflammatory cell activation. Furthermore, TMP significantly reduces IL-4 and IL-17 expression levels, diminishing inflammatory cell adhesion and infiltration in airways, which effectively alleviates airway inflammation and remodeling. This multi-targeted mechanism reduces inflammatory cell infiltration and cytokine overproduction while improving airway hyperresponsiveness. Additionally, it mitigates bronchospasm and decreases fibrotic changes, ultimately enhancing respiratory function in asthma patients. Crucially, TMP exhibits minimal adverse effects alongside its therapeutic efficacy, highlighting its potential as a safe complementary or alternative therapy. TMP's unique mechanistic profile and favorable safety data establish a robust foundation for novel asthma therapeutics. These advancements offer prospects for safer, more effective clinical options to improve patient outcomes.

Despite these insights, current research faces significant limitations. Preclinical studies predominantly rely on animal models, whose physiological and immunological differences from humans create translational uncertainties. Clinical evidence remains scarce, with most trials being small-scale and preliminary. The lack of large multicenter randomized controlled trials prevents definitive conclusions about TMP's efficacy and safety in human asthma management. While TMP shows preclinical promise and early clinical signals, it remains investigational. Rigorous human studies must now address three priorities: mechanistic validation in human pathways, optimized dosing regimens, and long-term safety assessments across diverse populations. Until such evidence emerges, cautious interpretation of TMP's clinical potential is essential. Future research should bridge this translational gap through coordinated preclinical-clinical efforts to fully realize TMP's therapeutic value in asthma.

## Methodology

The authors conducted systematic searches across PubMed, Web of Science, CNKI, Wanfang Data, and VIP databases from inception through May 2025. Search strategies combined keywords including “Tetramethylpyrazine”, “Asthma”, and “Pharmacological Mechanisms” using Boolean operators (AND/OR). Inclusion criteria prioritized peer-reviewed English/Chinese publications investigating TMP's effects on airway inflammation and remodeling in human asthma patients, animal models, and cellular studies. Following deduplication, eligible literature was categorized into inflammation regulation and remodeling modulation domains. These were further stratified by TMP's specific mechanistic pathways, forming the foundation for this comprehensive review.

## Abbreviations

This review addresses intricate molecular mechanisms requiring concise presentation. To enhance clarity and readability, the authors employ standardized abbreviations for recurrent specialized terms and complex nomenclature. The alphabetized abbreviations list below provides full definitions, which will be used directly in subsequent sections without repetitive elaboration (Table 1).

**Table 1:** Abbreviation table.

Abbreviation	Full Term	Abbreviation	Full Term
AC	Adenylate Cyclase	JNK	c-Jun N-terminal Kinase
AMPK	AMP-activated Protein Kinase	KDR	Kinase Insert Domain Receptor
AP-1	Activator Protein-1	MAPK	Mitogen-Activated Protein Kinase
ASMC	Airway Smooth Muscle Cells	MDA	Malondialdehyde
ATP	Adenosine Triphosphate	MMPs	Matrix Metalloproteinases
BA	Bronchial Asthma	MSCs	Mesenchymal Stem Cells
cAMP	cyclic Adenosine Monophosphate	NF-κB	Nuclear Factor-κB
CCL11/24	Eotaxin-1/2	NGF	Nerve Growth Factor
c-kit	Stem Cell Factor Receptor	ORMDL3	Orosomucoid-like 3
COX-2	Cyclooxygenase-2	RORγt	Retinoic acid receptor-related Orphan Receptor γt
CREB	cAMP-response element binding	PDE	Phosphodiesterase
CREM	cAMP-response element modulating	PI3K	Phosphatidylinositol 3-kinase
CTGF	Connective Tissue Growth Factor	PKA	Protein Kinase A
CVA	Cough Variant Asthma	PKB	Protein Kinase B
ECM	Extracellular Matrix	SAR1B	Secretion Associated Ras Related GTPase 1B
EGF	Epidermal Growth Factor	SCF	Stem Cell Factors
EMT	Epithelial-mesenchymal Transition	SIRT1	Silent Information Regulator 1
ERK	Extracellular Regulated Protein Kinases	SMC-A/Pi	Smooth Muscle Cell Area/Internal Perimeter
FGF	Fibroblast Growth Factor	STAT	Signal Transducer and Activator of Transcription
FOXP3	Forkhead Box Protein P3	T-bet	T-box expressed in T cells
GATA	GATA binding protein	TGF-β	Transforming Growth Factor-beta
GPCRs	G Protein-Coupled Receptors	Th	T helper
GSH-Px	Glutathione Peroxidase	TIMP	Tissue Inhibitor of Metalloproteinases
HGF	Hepatocyte Growth Factor	TLRs	Toll Like Receptors
HUVECs	Human Umbilical Vein Endothelial Cells	TMP	Tetramethylpyrazine
ICS	Inhaled Corticosteroids	TNF-α	Tumor Necrosis Factor-α
IκB	Inhibitor of Nuclear Factor κB	Treg	regulatory T
IFN-γ	Interferon-γ	UPR	Unfolded Protein Response
IL	Interleukin	VEGF	Vascular Endothelial Growth Factor
LABA	Long-Acting Beta2-Agonist	WA/Pi	Wall Area/Internal Perimeter
iNOS	Inducible Nitric Oxide Synthase	α-SMA	α-Smooth Muscle Actin
JAK	Janus Kinase		

## Funding

The article processing fee is funded by the Shandong Provincial Health Commission. Fei Yu received financial support from the Shandong Provincial Health Commission for the “Shandong Province Traditional Chinese Medicine Science and Technology Project” (Project Name: Research on the Mechanism of Ligustrazine and Its Derivatives in the Treatment of Chronic Refractory Asthma through the ORMDL3/ERK1/2/MMP-9/VEGF Signaling Pathway Based on the Blood-Activating and Stasis-Removing Method, n° M-20240301). The funding agency had no role in the review process, decision to publish, or preparation of the manuscript.

## CRediT authorship contribution statement

**Zichao Han:** Conceptualization, Methodology, Resources, Data curation, Writing – original draft, Writing – review & editing, Visualization. **Fei Yu:** Conceptualization, Methodology, Resources, Writing – original draft, Writing – review & editing, Supervision, Funding acquisition. **Zhen Ding:** Data curation, Writing – review & editing, Supervision. **Qinghua Liu:** Writing – review & editing, Project administration. **Xiangyi Zheng:** Writing – review & editing. **Lijuan Zhang:** Writing – review & editing. **Dong Li:** Writing – review & editing. **Chao Wang:** Writing – review & editing.

## Declaration of competing interest

The authors declare no conflicts of interest.

## References

[1] Dai C., Liu D., Qin C., Fang J., Cheng G., Xu C. (2025). Guben Kechuan granule attenuates bronchial asthma by inhibiting NF-κB/STAT3 signaling pathway-mediated apoptosis. J Ethnopharmacol.

[2] Li X., Song P., Zhu Y., Lei H., Chan K.Y., Campbell H. (2020). The disease burden of childhood asthma in China: a systematic review and meta-analysis. J Glob Health.

[3] Engelkes M., Baan E.J., de Ridder M.A.J., Svensson E., Prieto-Alhambra D., Lapi F. (2020). Incidence, risk factors and re-exacerbation rate of severe asthma exacerbations in a multinational, multidatabase pediatric cohort study. Pediatr Allergy Immunol.

[4] Reddel H.K., Bacharier L.B., Bateman E.D., Brightling C.E., Brusselle G.G., Buhl R. (2021). Global Initiative for Asthma Strategy 2021: executive summary and rationale for key changes. Eur Respir J.

[5] Braido F., Vlachaki I., Nikolaidis G.F., Tzelis D., Barouma I., Piraino A. (2025). Single inhaler with beclometasone, formoterol, and glycopyrronium versus triple therapies in adults with uncontrolled asthma: a systematic review and meta-analysis. Sci Rep.

[6] Vatti R.R., Ali F., Teuber S., Chang C., Gershwin M.E. (2014). Hypersensitivity reactions to corticosteroids. Clin Rev Allergy Immunol.

[7] Alves M.F., da Fonseca D.V., de Melo S.A.L., Scotti M.T., Scotti L., Dos Santos S.G. (2018). New therapeutic targets and drugs for the treatment of asthma. Mini Rev Med Chem.

[8] Chen Z., Zhang C., Gao F., Fu Q., Fu C., He Y. (2018). A systematic review on the rhizome of Ligusticum chuanxiong Hort. (Chuanxiong). Food Chem Toxicol.

[9] Ran X., Ma L., Peng C., Zhang H., Qin L.P. (2011). Ligusticum chuanxiong Hort: a review of chemistry and pharmacology. Pharm Biol.

[10] Lin J., Wang Q., Zhou S., Xu S., Tetramethylpyrazine Y.K. (2022). A review on its mechanisms and functions. Biomed Pharmacother.

[11] Yang S., Wu S., Dai W., Pang L., Xie Y., Ren T. (2021). Tetramethylpyrazine: a review of its antitumor potential and mechanisms. Front Pharmacol.

[12] Qian J., Xu Z., Zhu P., Meng C., Liu Y., Shan W. (2021). A derivative of piperlongumine and ligustrazine as a potential thioredoxin reductase inhibitor in drug-resistant hepatocellular carcinoma. J Nat Prod.

[13] Chen L., Liu T., Wang Q., Liu J. (2017). Anti-inflammatory effect of combined tetramethylpyrazine, resveratrol and curcumin in vivo. BMC Complement Altern Med.

[14] Zgorzynska E., Dziedzic B., Walczewska A. (2021). An overview of the Nrf2/ARE pathway and its role in neurodegenerative diseases. Int J Mol Sci.

[15] Luo X., Weng X., Bao X., Bai X., Lv Y., Zhang S. (2022). A novel anti-atherosclerotic mechanism of quercetin: competitive binding to KEAP1 via Arg483 to inhibit macrophage pyroptosis. Redox Biol.

[16] Guo M., Liu Y., Shi D. (2016). Cardiovascular actions and therapeutic potential of tetramethylpyrazine (Active Component Isolated from Rhizoma Chuanxiong): roles and mechanisms. Biomed Res Int.

[17] Meng Z., Chen H., Meng S. (2021). The roles of tetramethylpyrazine during neurodegenerative disease. Neurotox Res.

[18] Zhang Y., Ren P., Kang Q., Liu W., Li S., Li P. (2017). Effect of tetramethylpyrazine on atherosclerosis and SCAP/SREBP-1c signaling pathway in ApoE^-/-^ mice fed with a high-fat diet. Evid Based Complement Alternat Med.

[19] Liu X.M., Wang Y.B., Wu Q., Bian Z.R., Che X.W. (2018). Effects of ligustrazine on airway inflammation in A mouse model of neutrophilic asthma. Chin J Integr Med.

[20] Wang W-J, Yang L., Wang X-H, Li H-L (2004). Effect of ligustrazine on airway remodeling in asthmatic rats. Zhonghua Jie He He Hu Xi Za Zhi.

[21] Bunte K., Beikler T. (2019). Th17 Cells and the IL-23/IL-17 axis in the pathogenesis of periodontitis and Immune-mediated inflammatory diseases. Int J Mol Sci.

[22] Matia-Garcia I., Vadillo E., Pelayo R., Muñoz-Valle J.F., García-Chagollán M., Loaeza-Loaeza J. (2021). Th1/Th2 balance in young subjects: relationship with cytokine levels and metabolic profile. J Inflamm Res.

[23] Arellano G., Acuña E., Reyes L.I., Ottum P.A., De Sarno P., Villarroel L. (2017). Th1 and Th17 cells and associated cytokines discriminate among clinically isolated syndrome and multiple sclerosis phenotype. Front Immunol.

[24] Song Y.N., Lee J.W., Ryu H.W., Lee J.K., Oh E.S., Kim D.Y. (2023). Black ginseng extract exerts potentially anti-asthmatic activity by inhibiting the protein kinase Cθ-mediated IL-4/STAT6 signaling pathway. Int J Mol Sci.

[25] Tumes D.J., Papadopoulos M., Endo Y., Onodera A., Hirahara K., Nakayama T. (2017). Epigenetic regulation of T-helper cell differentiation, memory, and plasticity in allergic asthma. Immunol Rev.

[26] Kim S.H., Kim B.K., Lee Y.C. (2011). Antiasthmatic effects of hesperidin, a potential Th2 cytokine antagonist, in a mouse model of allergic asthma. Mediat Inflamm.

[27] Xiong G.H., Liu S.Y., Gao J.L., Wang S.M. (2016). Naringin protects ovalbumin-induced airway inflammation in a mouse model of asthma. Inflammation..

[28] Caminati M., Pham D.L., Bagnasco D., Canonica G.W. (2018). Type 2 immunity in asthma. World Allergy Organ J.

[29] Xiong L., Fang Z.Y., Tao X.N., Bai M., Feng G. (2007). Effect and mechanism of ligustrazine on Th1/Th2 cytokines in a rat asthma model. Am J Chin Med.

[30] Yan Y.F. (2009). Disruption of Th1/Th2 balance in peripheral blood of asthmatic children and the interventional effect of ligustrazine. Strait Pharm J.

[31] Wooldridge A.L., Clifton V.L., Moss T.J.M., Lu H., Jamali M., Agostino S. (2019). Maternal allergic asthma during pregnancy alters fetal lung and immune development in sheep: potential mechanisms for programming asthma and allergy. J Physiol.

[32] Katoh S. (2022). Critical involvement of CD44 in T helper type 2 cell-mediated eosinophilic airway inflammation in a mouse model of acute asthma. Front Immunol.

[33] Kumar S, Lanckacker E, Dentener M, Bracke K, Provoost S, De Grove K, et al. Aggravation of allergic airway inflammation by cigarette smoke in mice is CD44-dependent. PLoS One. 2016;11(3):e0151113. 10.1371/journal.pone.0151113 PMC4801229 26999446

[34] Li L., Yang L., Tang H., Jin R. (2005). Experimental study on the intervention mechanism of ligustrazine on CD44 in lung tissue of asthmatic rats. Modern Med J.

[35] Kargar M., Torabizadeh M., Purrahman D., Zayeri Z.D., Saki N. (2023). Regulatory factors involved in Th17/Treg cell balance of immune thrombocytopenia. Curr Res Transl Med.

[36] Santamaria J.C., Borelli A., Irla M. (2021). Regulatory T cell heterogeneity in the Thymus: impact on their functional activities. Front Immunol..

[37] Luo A., Leach S.T., Barres R., Hesson L.B., Grimm M.C., Simar D. (2017). The microbiota and epigenetic regulation of T helper 17/regulatory T cells: in search of a balanced immune system. Front Immunol.

[38] Huang L., Wang M., Yan Y., Gu W., Zhang X., Tan J. (2018). OX40L induces helper T cell differentiation during cell immunity of asthma through PI3K/AKT and P38 MAPK signaling pathway. J Transl Med.

[39] Ji N.F., Xie Y.C., Zhang M.S., Zhao X., Cheng H., Wang H. (2014). Ligustrazine corrects Th1/Th2 and Treg/Th17 imbalance in a mouse asthma model. Int Immunopharmacol.

[40] Pan R., Zhou M., Zhong Y., Xie J., Ling S., Tang X. (2019). The combination of Astragalus membranaceus extract and ligustrazine to improve the inflammation in rats with thrombolytic cerebral ischemia. Int J Immunopathol Pharmacol.

[41] Sharma P., Penn R.B. (2021). Can GPCRs Be targeted to control inflammation in asthma?. Adv Exp Med Biol.

[42] De Cesare D., Sassone-Corsi P. (2000). Transcriptional regulation by cyclic AMP-responsive factors. Prog Nucleic Acid Res Mol Biol.

[43] Lee J., Kim T.H., Murray F., Li X., Choi S.S., Broide D.H. (2015). Cyclic AMP concentrations in dendritic cells induce and regulate Th2 immunity and allergic asthma. Proc Natl Acad Sci U S A.

[44] Wang Y.J., Zou Y.Y., Gao H.W., Chen M., Wang T.S. (2022). Effect of ligustrazine on Gαs/cAMP/PKA signal activity and airway inflammation in asthmatic rats. Chin Pharmacol Bull.

[45] Wang Y., Zhu H., Tong J., Li Z. (2022). Ligustrazine inhibits lung phosphodiesterase activity in a rat model of allergic asthma. Comput Math Methods Med.

[46] Haak A.J., Ducharme M.T., Diaz Espinosa A.M., Tschumperlin D.J. (2020). Targeting GPCR signaling for idiopathic pulmonary fibrosis therapies. Trends Pharmacol Sci.

[47] Hu R., Wang M.Q., Ni S.H., Wang M., Liu L.Y., You H.Y. (2020). Salidroside ameliorates endothelial inflammation and oxidative stress by regulating the AMPK/NF-κB/NLRP3 signaling pathway in AGEs-induced HUVECs. Eur J Pharmacol.

[48] Li J., Zhong L., Wang F., Zhu H. (2017). Dissecting the role of AMP-activated protein kinase in human diseases. Acta Pharm Sin B.

[49] Xu C., Song Y.L., Jiang J.Z., Wang Z.G., Piao Y.H., Li L.C. (2021). Ligustrazine reduces allergic airway inflammation and oxidative stress through AMPK/NF-κB and Nrf-2/HO-1 pathways. Immunol J.

[50] Zhu L., Bi Y., Liang T., Zhang P., Xiao X., Yu T. (2025). Ginkgetin delays the progression of osteoarthritis by inhibiting the NF-κB and MAPK signaling pathways. J Orthop Surg Res.

[51] Liang L., Gu X., Shen H.J., Shi Y.H., Li Y., Zhang J. (2021). Chronic intermittent hypoxia reduces the effects of glucosteroid in asthma via activating the p38 MAPK signaling pathway. Front Physiol.

[52] Widmann C., Gibson S., Jarpe M.B., Johnson G.L. (1999). Mitogen-activated protein kinase: conservation of a three-kinase module from yeast to human. J Physiol Rev.

[53] Mei Y.T., Huang C.P., Hu K., Zhao P.Z., Xiao J.X., Zhao J.J. (2011). Effect of ligustrazine on the expression of p38 mitogen-activated protein inase p38MAPK and tryptase in asthmatic mice. J Clin Pulm Med.

[54] Wei Y., Liu J., Zhang H., Du X., Luo Q., Sun J. (2016). Ligustrazine attenuates inflammation and the associated chemokines and receptors in ovalbumine-induced mouse asthma model. Environ Toxicol Pharmacol.

[55] Higham A., Singh D. (2021). Dexamethasone and p38 MAPK inhibition of cytokine production from human lung fibroblasts. Fundam Clin Pharmacol.

[56] Perng D.W., Wu Y.C., Tsai C.C., Su K.C., Liu L.Y., Hsu W.H. (2008). Bile acids induce CCN2 production through p38 MAP kinase activation in human bronchial epithelial cells: a factor contributing to airway fibrosis. Respirology.

[57] Xie M., Liu T., Yin J., Liu J., Yang L., Li T. (2024). Kechuanning gel plaster exerts anti-inflammatory and immunomodulatory effects on ovalbumin-induced asthma model rats via ERK pathway. Comb Chem High Throughput Screen.

[58] Chen R.H., Ji A.B., Wang F. (2005). Effect of tetramethylpyrazine on the expression of C-fos in asthmatic rats. J Nantong Univ (Med Sci).

[59] Zhang Y., Li T.Z., Liu C.T., Wang D.L. (2008). Research of the effects of simulated microgravity on C-fos of the pulmonary tissues in rats and the ligustrazine counter its function. Acad J Chin PLA Med Sch.

[60] Tayel S.I., El-Hefnway S.M., Abd El Gayed E.M., Abdelaal G.A. (2017). Association of stem cell factor gene expression with severity and atopic state in patients with bronchial asthma. Respir Res.

[61] Mak A.C.Y., Sajuthi S., Joo J., Xiao S., Sleiman P.M., White M.J. (2020). Lung function in African American children with asthma is associated with novel regulatory variants of the KIT Ligand KITLG/SCF and gene-by-air-pollution interaction. Genetics.

[62] Liu J., Yin K.S., Bian T., Xue Y.F., Hu Z.G. (2007). Effect of ligustrazine inhibition on the expression of stem cell factor (SCF) in mouse asthmatic models. J Nanjing Med Univ (Nat Sci).

[63] Johansson M.W., Grill B.M., Barretto K.T., Favour M.C., Schira H.M., Swanson C.M. (2020). Plasma P-selectin is inversely associated with lung function and corticosteroid responsiveness in asthma. Int Arch Allergy Immunol..

[64] Woollard K.J. (2010). Chin-Dusting J. P-selectin antagonism in inflammatory disease. Curr Pharm Des.

[65] Bi J., Hu Y., Peng Z., Liu H., Fu Y. (2018). Changes and correlations of serum interleukins, adhesion molecules and soluble E-selectin in children with allergic rhinitis and asthma. Pak J Med Sci.

[66] Tang H., Yang L., Li L. (2006). The effect of ligustrazine on P-selectin expression in serum of asthmatic rats. Mod Med J.

[67] Ye J.M. (2008). The regulatory effect of ligustrazine on P-selectin in peripheral blood of asthmatic children. Anhui Med Pharm J.

[68] Yan S.N. (2009). The regulatory effects of ligustrazine on P-selectin and soluble E-selectin in peripheral blood of asthma patients. Mod Pract Med.

[69] Wang Y., Zhu H., Tong J., Li Z. (2016). Ligustrazine improves blood circulation by suppressing platelet activation in a rat model of allergic asthma. Environ Toxicol Pharmacol.

[70] Domvri K., Tsiouprou I., Bakakos P., Steiropoulos P., Katsoulis K., Kostikas K. (2025). Effect of mepolizumab in airway remodeling in patients with late-onset severe asthma with an eosinophilic phenotype. J Allergy Clin Immunol.

[71] Rønnow S.R., Sand J.M.B., Staunstrup L.M., Bahmer T., Wegmann M., Lunding L. (2022). A serological biomarker of type I collagen degradation is related to a more severe, high neutrophilic, obese asthma subtype. Asthma Res Pract.

[72] Listyoko A.S., Okazaki R., Harada T., Takata M., Morita M., Ishikawa H. (2024). β-tocotrienol decreases PDGF-BB-induced proliferation and migration of Human airway smooth muscle cells by inhibiting RhoA and reducing ROS production. Pharmaceuticals (Basel).

[73] Li L., Kong L., Fang X., Jiang C., Wang Y., Zhong Z. (2009). SH2-B beta expression in alveolar macrophages in BAL fluid of asthmatic guinea pigs and its role in NGF-TrkA-mediated asthma. Respirology.

[74] Hsieh A., Yang C.X., Al-Fouadi M., Nwozor K.O., Osei E.T., Hackett T.L. (2023). The contribution of reticular basement membrane proteins to basal airway epithelial attachment, spreading and barrier formation: implications for airway remodeling in asthma. Front Med (Lausanne).

[75] Ojiaku C.A., Yoo E.J., Jr P.R.A. (2017). Transforming growth factor β1 function in airway remodeling and hyperresponsiveness. The missing link?. Am J Respir Cell Mol Biol.

[76] Hu H.H., Chen D.Q., Wang Y.N., Feng Y.L., Cao G., Vaziri N.D. (2018). new insights into TGF-β/smad signaling in tissue fibrosis. Chem Biol Interact.

[77] Radajewski K., Kalińczak-Górna P., Zdrenka M., Antosik P., Wierzchowska M., Grzanka D. (2021). Short term pre-operative oral corticosteroids-tissue remodeling in chronic rhinosinusitis with nasal polyps. J Clin Med.

[78] Yang N., Zhang H., Cai X., Shang Y. (2018). Epigallocatechin-3-gallate inhibits inflammation and epithelial-mesenchymal transition through the PI3K/AKT pathway via upregulation of PTEN in asthma. Int J Mol Med.

[79] Shi Y.X., Dai X., Wang L.J., Wu J., Jiang S., Yang X.Q. (2019). Effect of ligustrazine on airway inflammation and airway remodeling in asthmatic mice by regulating TGF-β/smad signaling pathway. Drugs Clin.

[80] Matoba A., Matsuyama N., Shibata S., Masaki E., Sr E.C.W., Mizuta K. (2018). The free fatty acid receptor 1 promotes airway smooth muscle cell proliferation through MEK/ERK and PI3K/akt signaling pathways. Am J Physiol Lung Cell Mol Physiol.

[81] Sun Y.Y., Ren J.H., Yang B. (2021). Effects of ligustrazine on transforming growth factor-β family proteins expression in airway smooth muscle tissue in rats with cough variant asthma. Chin J Clin Pharmacol.

[82] Wu S.M., Wu Y.J., Liu L., Cai R.P., Yu Y.J. (2009). Effect of Baicalin and Ligustrazine on airway wall remodeling and underlying mechanism in asthmatic rats. Acta Med Univ Sci Technol Huazhong.

[83] Hackett T.L. (2012). Epithelial-mesenchymal transition in the pathophysiology of airway remodelling in asthma. Curr Opin Allergy Clin Immunol.

[84] Zhong X., Li M. (2022). Molecular mechanism of airway epithelial cells involved in airway remodeling through epithelial mesenchymal transition in asthma. Int J Pediatr.

[85] Kim K.K., Kugler M.C., Wolters P.J., Robillard L., Galvez M.G., Brumwell A.N. (2006). Alveolar epithelial cell mesenchymal transition develops in vivo during pulmonary fibrosis and is regulated by the extracellular matrix. Proc Natl Acad Sci U S A.

[86] Zhang B., Feng X., Tian L., Xiao B., Hou L., Mo B. (2025). Epithelial-mesenchymal transition in asthma: its role and underlying regulatory mechanisms. Front Immunol.

[87] Shi M.R., Zhang N.Z., Chu W.F., Jin C.C. (2024). Research progress on the mechanism of intervention of Chinese herbs on epithelial-mesenchymal transition in bronchial asthma. J Shanxi Univ Chin Med.

[88] Montero P., Milara J., Roger I., Cortijo J. (2021). Role of JAK/STAT in interstitial Lung diseases; molecular and cellular mechanisms. Int J Mol Sci.

[89] Ou H.P., Wu Q.H., Yuan D., Lin H., He C.R., Li D. (2023). Effect of Taohong Siwu Decoction on apoptosis and EMT of pulmonary fibrosis model rats by JAK2/STAT3 signaling pathway. Chin Pharmacol Bull.

[90] Almacioglu M., Keskin O., Ozkars M.Y., Balci S.O., Kucukosmanoglu E., Pehlivan S. (2023). Association of childhood asthma with Gasdermin B (GSDMB) and oromucoid-like-3 (ORMDL3) genes. North Clin Istanb.

[91] Cheng Q., Shang Y. (2018). ORMDL3 may participate in the pathogenesis of bronchial epithelial‑mesenchymal transition in asthmatic mice with airway remodeling. Mol Med Rep.

[92] Miller M., Tam A.B., Cho J.Y., Doherty T.A., Pham A., Khorram N. (2012). ORMDL3 is an inducible lung epithelial gene regulating metalloproteases, chemokines, OAS, and ATF6. Proc Natl Acad Sci U S A.

[93] Yu F., Sun Y., Yu J., Ding Z., Wang J., Zhang L. (2017). ORMDL3 is associated with airway remodeling in asthma via the ERK/MMP-9 pathway. Mol Med Rep.

[94] Wang H., Liu Y., Shi J., Cheng Z.. (2019). ORMDL3 knockdown in the lungs alleviates airway inflammation and airway remodeling in asthmatic mice via JNK1/2-MMP-9 pathway. Biochem Biophys Res Commun.

[95] Li Y., Li X., Zhou W., Yu Q., Lu Y. (2020). ORMDL3 modulates airway epithelial cell repair in children with asthma under glucocorticoid treatment via regulating IL-33. Pulm Pharmacol Ther.

[96] Ding Z., Yu F., Wan J.H. (2021). Effect of ligustrazine on the expression of orosomucoid 1-like protein 3 in bronchial epithelial cells. J Qingdao Univ (Med Sci).

[97] Yang L.J., Sui S.X., Zheng Q.H., Wang M. (2024). circUQCRC2 promotes asthma progression in children by activating the VEGFA/NF-κB pathway by targeting miR-381-3p. Kaohsiung J Med Sci.

[98] Ma D., Muñoz X., Ojanguren I., Romero-Mesones C., Soler-Segovia D., Varona-Porres D. (2024). Increased TGFβ1, VEGF and IFN-γ in the sputum of severe asthma patients with bronchiectasis. Arch Bronconeumol.

[99] Feng Z., Jia C., Han B., Chen X., Mei J., Qiao S. (2025). The causal role of immune cell phenotypes and inflammatory factors in childhood asthma: evidence from mendelian randomization. Pediatr Pulmonol.

[100] Nasser M., Fahmey S., Geogry D., Taha G.E. (2019). Expression of serum MicroRNAs 221, 222, 15a and level of VEGF-A in children with bronchial asthma. Egypt J Immunol.

[101] Yan G.H., Hou Q.Z., Chen F.H., Yan Z.H. (2010). TMP control VEGF and iN0S expression of asthma rats in airway to affect airway remodeling. J Xiangnan Univ (Med Sci).

[102] Cheng M., Li Y., Wu J., Nie Y., Li L., Liu X. (2008). IL-8 induces imbalances between nitric oxide and endothelin-1, and also between plasminogen activator inhibitor-1 and tissue-type plasminogen activator in cultured endothelial cells. Cytokine.

[103] Faiz A., Harkness L.M., Tjin G., Bernal V., Horvatovich P., James A. (2021). Angiogenic regulatory influence of extracellular matrix deposited by resting state asthmatic and non-asthmatic airway smooth muscle cells is similar. J Cell Mol Med.

[104] Gao F., Chiu S.M., Motan D.A., Zhang Z., Chen L., Ji H.L. (2016). Mesenchymal stem cells and immunomodulation: current status and future prospects. Cell Death Dis.

